# Hypokalemia associated with pseudo-Cushing’s syndrome and magnesium deficiency induced by chronic alcohol abuse

**DOI:** 10.1007/s13730-018-0315-4

**Published:** 2018-02-15

**Authors:** Masafumi Kurajoh, Keiko Ohsugi, Miki Kakutani-Hatayama, Takuhito Shoji, Hidenori Koyama

**Affiliations:** Division of Diabetes, Endocrinology and Metabolism, Department of Internal Medicine, Hyogo College of Medicine, 1-1 Mukogawa-cho, Nishinomiya, Hyogo 663-8501 Japan

**Keywords:** Hypokalemia, Magnesium deficiency, Pseudo-Cushing syndrome, Magnesium administration

## Abstract

Hypokalemia and hypomagnesemia are frequently observed in patients with chronic alcoholism. However, the involvement of deranged cortisol regulation in patients with those conditions has not been reported. A 63-year-old Japanese male with chronic alcoholism was referred to the Department of Diabetes, Endocrinology and Metabolism for examination and treatment of hypokalemic periodic paralysis. Laboratory findings showed hypokalemia (2.3 mmol/l), as well as a high level of urinary excretion of potassium and hypomagnesemia (1.2 mg/dl), whereas urinary excretion of magnesium was undetectable. Potassium infusion treatment recovered that level in serum to 4.1 mmol/l, though it decreased to 2.2 mmol/l following discontinuation. A dexamethasone suppression test and urinary cortisol level showed corticotropin-dependent hypercortisolemia. However, gadolinium-enhanced MRI revealed no evidence of pituitary adenoma. The patient recovered from hypokalemia following an administration of magnesium in addition to potassium, which was accompanied by potassium over-excretion improvement. After being discharged, serum potassium level was maintained within a normal range with only magnesium infusion treatment. Furthermore, alcohol intake was reduced from 160 to 20 g/day and an endocrinological re-examination after that restriction showed normal cortisol regulation. The patient was diagnosed with pseudo-Cushing’s syndrome induced by alcohol abuse. Serum potassium level was maintained within a normal range even after discontinuation of magnesium supplementation. Our findings in this case indicate that pseudo-Cushing’s syndrome in conjunction with hypomagnesemia may be involved in development of hypokalemia in patients with chronic alcoholism.

## Introduction

Patients with chronic alcoholism are frequently affected by hypokalemia and hypomagnesemia [1]. Chronic alcoholism can also lead to low dietary intake, vomiting, and diarrhea, resulting in malabsorption of magnesium and potassium. In addition, urinary over-secretion of potassium can be attributed to hypokalemia in patients with alcohol abuse [2]. Impairment of Na–K-ATPase induced by hypomagnesemia, which causes a decrease in cellular uptake of potassium, is thought to induce potassium over-secretion into urine [3], while another recent report suggested that intracellular magnesium inhibits tubular renal outer medullary K^+^ (ROMK) channel-mediated potassium secretion into urine [4]. However, magnesium deficiency alone does not necessarily induce hypokalemia, as additional factors such as hyperaldosteronism are thought to be necessary for exacerbation of potassium wasting [4].

Here, we report a case of hypokalemia and hypomagnesemia associated with pseudo-Cushing’s syndrome induced by chronic alcoholism, in which hypokalemia was improved by magnesium supplementation. Following normalization of pseudo-Cushing’s syndrome with mitigation of excessive alcohol consumption, magnesium supplementation was no longer necessary to maintain serum potassium level in this patient. Our findings suggest the potential involvement of pseudo-Cushing’s syndrome in hypokalemia and effects of magnesium supplementation in patients with alcohol abuse.

## Case report

**Fig. 1:**
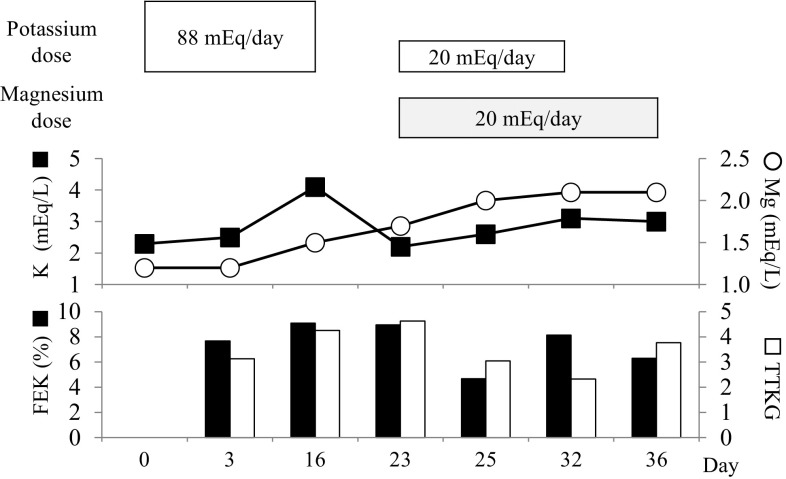
Clinical course of potassium metabolism and serum magnesium levels. *FEK* fractional excretion of potassium, *TTKG* trans-tubular potassium gradient

A 63-year-old Japanese man was admitted to the Cardiovascular Division of the Hyogo College of Medicine Hospital on an emergency basis for recurrent hypokalemic periodic paralysis (Day 0). Upon admission, serum potassium was 2.3 mmol/l, while other laboratory findings are shown in Table 1. Those findings revealed that sodium was at the upper limit of normal, serum magnesium was lower than normal, and urine magnesium was not detectable. An infusion of potassium (88 mmol/day) was started and continued for 16 days, after which serum potassium recovered to 4.1 mmol/l (Fig. 1). At 7 days after discontinuation of potassium infusion (Day 23), the potassium level in serum was again decreased to 2.2 mmol/l. Furthermore, the values for fractional excretion of potassium (FEK) and trans-tubular potassium gradient (TTKG) were 8.95 and 4.26%, respectively, which were higher in spite of the low serum potassium level. The patient was referred to the Division of Diabetes, Endocrinology and Metabolism for further examinations and treatment for profound hypokalemia.

**Table 1:** Laboratory findings

Variables	On admission	After transfer to our division	At discharge	Normal range
Blood cell counts				
WBC (/µl)	5450	6460	4600	4000–9000
Segmentation (%)	69	73.1	62.4	38.0–58.0
Lymphocytes (%)	19.4	20.4	19.3	26.4–47.0
Eosinophils (%)	2.0	1.4	12.6	2.0–7.0
Hemoglobin (g/dl)	9.3	10.5	11.9	13.0–17.0
Platelets (10^4^/µl)	27.7	40.1	30	15.0–35.0
Blood biochemical analysis				
Total bilirubin (mg/dl)	0.9	1.0	1.1	0.2–1.2
AST (IU/l)	13	26	13	13–33
ALT (IU/l)	8	21	23	8–42
γ-GTP (IU/l)	32	37	33	1–58
Total protein (g/dl)	5.9			6.6–8.7
Albumin (g/dl)	3.2	3.7	3.8	3.7–4.7
CK (IU/l)	318	22	42	62–287
Creatinine (mg/dl)	0.75	0.79	1.01	0.36–1.06
Magnesium (mg/dl)	1.2	1.5	1.8	1.9–2.5
Sodium (mEq/l)	146	142	143	138–146
Potassium (mEq/l)	2.3	4.1	3.9	3.6–4.9
Chloride (mEq/l)	106	113	105	99–109
FPG (mg/dl)	99	81	81	77–109
Blood endocrinological analysis				
Free T4 (ng/dl)	1.03	1.07		0.9–1.7
TSH (µIU/ml)	1.63	2.23		0.5–5.0
ACTH (pg/ml)	56.6	45.1		7.2–63.3
Cortisol (µg/dl)	15.3	15.1		4.0–18.3
PRA (ng/ml/h)	0.2	1.1		0.3–2.9
PAC (pg/ml)	45.1	57.7		29.9–159
Urine analysis				
Cortisol (µg/day)	153	147		11.2–80.3
Aldosterone (µg/day)		1.4		0.0–10.0
Magnesium (mg/day)	ND	ND		

*ND* not detectable, *PRA* plasma renin activity, *PAC* plasma aldosterone concentration

A physical examination showed a body height of 173 cm, weight of 65 kg, and blood pressure at 140/76 mmHg. The patient had cushingoid features, such as proximal muscle weakness and mild truncal obesity, but not a moon face or buffalo hump. He had been drinking approximately 160 g of alcohol per day for more than 40 years and ate only limited amounts of soy food, seafood, seaweed, vegetables, and fruits. Diarrhea occasionally occurred. Medical history included several instances of hypokalemic periodic paralysis that required emergency transport to our hospital, even though oral potassium supplementation had been given. At those times, recovery from hypokalemia and periodic paralysis was obtained by potassium infusion. He had also been treated for hypertension and hyperuricemia. In addition, the patient had smoked 40 cigarettes/per day for more than 40 years. There was no family history of periodic paralysis, while his brother had hypertension.

Following transfer to our division, laboratory results showed that the urine cortisol level (147 µg/day) was greater than the normal range (11.2–80.3 µg/day) (Table 1). A dexamethasone suppression test (0.5 mg) failed to suppress serum ACTH and cortisol levels (41.3 pg/ml and 9.2 µg/dl, respectively) (Table 2). The patient was diagnosed with ACTH-dependent Cushing’s syndrome, though gadolinium-enhanced MRI revealed no evidence of a pituitary adenoma. We speculated that hypercortisolemia accentuated hypomagnesemia-mediated tubular over-secretion of potassium in this case, and daily infusions of magnesium (20 mmol/day) and potassium (20 mmol/day) were initiated (day 23). The decreases in serum potassium level, and enhanced TTKG and FEK were gradually recovered by day 32. After stopping the infusion of magnesium on day 36, serum potassium, and TTKG and FEK levels were maintained, and the patient was discharged. After returning home, he received weekly infusions of magnesium (20 mmol/week) for 5 months on an outpatient basis and serum potassium was maintained within a normal range.

**Table 2:** Diurnal fluctuation of plasma ACTH/cortisol, urinary cortisol, and dexamethasone (Dex) suppression test, before and after reduction of alcohol intake

	8:00	16:00	23:00	0.5 mg Dex	8 mg Dex
Initial admission to our division					
ACTH (pg/ml)	56.6	45.1	20.1	41.3	NA
Cortisol (µg/dl)	15.3	13.4	6.7	9.2	NA
U-Cortisol (µg/day)	147				
Six months after reduction of alcohol intake					
ACTH (pg/ml)	51.1	26.7	16.8	25.4	≤ 2.0
Cortisol (µg/dl)	27.1	12.6	5.2	4.4	1.8
U-Cortisol (µg/day)	43.9				

*NA* not assessed

We thought that alcohol abuse may have led to deranged cortisol regulation (pseudo-Cushing’s syndrome) in this patient and alcohol intake was restricted to 20 g/day after discharge. After 6 months of reduced intake, he underwent re-examinations for determining potential recovery of deranged cortisol regulation. Urinary cortisol was within a normal range (43.9 µg/day), and a 0.5-mg dexamethasone suppression test showed normal ACTH and cortisol regulation (Table 2). Furthermore, blood pressure was controlled (126/86 mmHg), and proximal muscle weakness and mild truncal obesity had disappeared. Thus, the reduction of alcohol intake appeared to have restored deranged cortisol regulation and we made a diagnosis of pseudo-Cushing’s syndrome due to chronic alcohol abuse. The magnesium infusions were discontinued and serum potassium was maintained in a normal range for 5 months. Thereafter, the patient gradually increased alcohol intake to approximately 160 g/day and again serum potassium dropped to 2.8 mmol/l, which was improved to 5.2 mmol/l after 1 month of magnesium infusion therapy (20 mmol/week).

## Discussion

Although alcohol abuse is frequently associated with hypokalemia and hypomagnesemia [4, 5], no involvement of deranged cortisol regulation in that process has been shown. Findings for the present patient suggested that ACTH-dependent hypercortisolism induced by alcohol abuse (pseudo-Cushing’s syndrome) profoundly contributed to the pathophysiology of hypokalemia associated with hypomagnesemia. Magnesium supplementation improved tubular potassium over-secretion and hypokalemia, while improvement of pseudo-Cushing’s syndrome following a reduction of alcohol intake was associated with maintenance of serum potassium level without magnesium supplementation.

Hypokalemia and hypomagnesemia are frequently observed in patients with chronic alcoholism [2]. Hypomagnesemia occurring in such individuals can be caused by renal over-wasting and/or gastrointestinal malabsorption of magnesium in relation to vomiting or diarrhea [5], though the mechanism of renal magnesium wasting associated with chronic alcoholism remains unknown. On the other hand, no evidence of hypercortisolism-induced hypomagnesemia has been reported. The undetectable level of urinary magnesium in our patient indicates a gastrointestinal cause and his low intake of soy products, seafood, and seaweed, and frequent diarrhea potentially attributed to gastrointestinal malabsorption of magnesium.

Hypokalemia in association with alcoholism can also be attributed to gastrointestinal and renal causes. In the present patient, the low intake of potassium, commonly found in vegetables and fruits, which he rarely consumed, and occasional diarrhea contributed to a low level of gastrointestinal potassium absorption. Furthermore, the high urinary excretion of potassium, in spite of the presence of hypokalemia, indicates that renal over-wasting of potassium into urine was the primary cause. Indeed, tubular potassium over-secretion has been reported to occur in 75% of patients with chronic alcoholism [2].

Magnesium deficiency contributes to hypokalemia in patents with alcohol abuse [6]. Such deficiency impairs magnesium-activated ATPase-dependent cell membrane cation pumps, resulting in a decrease in cellular potassium uptake and increase in urinary potassium excretion [3]. Repletion of magnesium has been shown to improve hypokalemia in patients with alcoholism [5] and thiazide treatment [7]. In addition, hypokalemia and over-excretion of potassium was reported to be improved in patients with Bartter syndrome after magnesium supplementation [8].

A recent study suggested involvement of the ROMK channel, which secretes potassium into the luminal side via depolarization of the luminal membrane in response to epithelial sodium channel (ENac)-mediated sodium reabsorption. In hyperaldosteronism, tubular potassium secretion via the ROMK is increased in accordance with accelerated sodium uptake via the ENac. Importantly, magnesium deficiency is known to accelerate the function of ROMK [4]. Therefore, it is feasible that ROMK-mediated over-wasting of potassium is accelerated by mineral corticoid action in pseudo-Cushing’s status and by magnesium deficiency in patients with alcohol abuse. These imply that restriction of alcohol intake might not only ameliorate hypercortisolemia but also improve hypomagnesemia, resulting in improvement of hypokalemia.

The clinical and biochemical features of Cushing’s syndrome and alcohol-induced pseudo-Cushing’s syndrome are similar and not distinguishable [9, 10]. Therefore, it is necessary to consider alcohol-induced pseudo-Cushing’s syndrome in patients with chronic alcoholism who have clinical features associated with Cushing’s syndrome. Biochemical derangements in the present patient included insufficient suppression after low-dose dexamethasone and increased 24-h urinary free cortisol, which are the most frequent abnormalities occurring in relation to pseudo-Cushing’s status [9, 10]. As shown in this case, resolution of clinical and biochemical disturbances often occurs after alcohol withdrawal.

It is difficult to completely negate the possibility that the lifestyle factors (alcohol abuse, low intake of potassium) of our patient and not pseudo-Cushing’s status were the primary cause of hypokalemia associated with hypomagnesemia. Nevertheless, hypokalemia and urinary over-secretion of potassium continued for more than 3 weeks during hospitalization, implying that those factors were not the sole contributors.

## Conclusion

We report findings showing that ACTH-dependent hyperadrenocortisolism induced by alcohol abuse in the present patient (pseudo-Cushing’s syndrome) profoundly affected the pathophysiology of hypokalemia associated with hypomagnesemia.
